# Disinfection through Advance Oxidation Processes: Optimization and Application on Real Wastewater Matrices

**DOI:** 10.3390/toxics10090512

**Published:** 2022-08-30

**Authors:** Pablo Blanco-Canella, Gabriela Lama, Mª Angeles Sanromán, Marta Pazos

**Affiliations:** CINTECX, Department of Chemical Engineering, Universidade de Vigo, Campus As Lagoas-Marcosende, 36310 Vigo, Spain

**Keywords:** disinfection, *E. coli*, Fenton, Fenton-like, wastewater matrices

## Abstract

Disinfection is an essential and significant process for water treatment to protect the environment and human beings from pathogenic infections. In this study, disinfection through the generation of hydroxyl (Fenton process (FP)) and sulfate (Fenton-like process (FLP)) radicals was validated and optimized. The optimization was carried out in synthetic water through an experimental design methodology using the bacteria *Escherichia coli* as a model microorganism. Different variables were evaluated in both processes: precursor concentration (peroxymonosulfate (PMS) and H_2_O_2_), catalyst concentration (Fe^+2^), and pH in the Fenton process. After that, the optimized conditions (FP: 132.36 mM H_2_O_2_, 0.56 mM Fe^+2^ and 3.26 pH; FLP: 3.82 mM PMS and 0.40 mM Fe^+2^) were applied to real matrices from wastewater treatment plants. The obtained results suggest that both processes are promising for disinfection due to the high oxidant power of hydroxyl and sulfate radicals.

## 1. Introduction

In urban cities, wastewater treatment plants (WWTPs) are essential to remediate water and give it a second life. For this reason, sewer systems are utilized globally to collect sewage, contribute to pollution control, and improve human health. However, their technology is not strong enough to remove all the pathogens from the water, so many of them are released into the environment. Although this is done in low concentrations, it is still enough to cause infections [1]. Nowadays, disinfection is an essential and significant process for water treatment to protect the environment and human beings from pathogenic infections [2]. There are a wide variety of techniques and methods, such as UV, chlorination, radiation, or coagulation. However, some of these technologies have drawbacks because, during the disinfection process, some carcinogenic disinfection by-products are released and some pathogens are resistant [3,4].

In recent days, advanced oxidation processes (AOPs), which are mainly based on the formation of highly oxidative species (mainly HO^•^ or SO_4_^•−^), have attracted great interest as disinfection treatments because they have proved to be effective for bacteria inactivation [5,6]. Furthermore, they are environmentally friendly and can non-selectively destroy most organic and organometallic contaminants until their complete mineralization; that is, their conversion into CO_2_, H_2_O, and inorganic species [7,8,9]. Furthermore, the inactivation of microorganisms’ pathogens is achieved through the membrane, proteins, lipids, enzymes, DNA, and RNA damage [4].

In this study, the attention focuses on two AOPs, the Fenton process (FP) based on the traditional Fenton reaction where hydroxyl radicals are produced, and Fenton-like processes (FLP) in which the generation of free radicals as sulfate radicals are proposed as an alternative to hydroxyl radicals [10]. The FP is based on the hydrogen peroxide decomposition with the presence of Fe^+2^ to the formation of radicals through equation 1. The organic matter could be oxidized by hydrogen abstraction or by hydroxyl addition of those hydroxyl radicals [11]. Apart from the oxidant and the catalyst, there is another variable in the process: the pH of the reaction, which will influence the process. Low pH (around 3) is necessary to accomplish the treatment.

(1) $$H_{2}O_{2}+{\mathrm{Fe}}^{2+}\to {\;\mathrm{Fe}}^{3+}+\mathrm{HO}\cdot +{\mathrm{OH}}^{-}$$

In recent years, several concentration ranges of oxidants have been tested for FP; however, a high concentration (>20 mM) of H_2_O_2_ is necessary in order to react with the cell membrane directly to increase its permeability and damage all the macromolecules [4,12]. In addition, most of the studies were performed using simple matrices (distilled water), without the presence of compounds present in the real wastewater [13,14] which can reduce the efficiency of the disinfection interfering as a scavenger to the radicals formed. Thus, there is a need to increase the knowledge related to the disinfection under conditions similar to real applications.

On the other hand, in the FLP sulfate, radicals can be generated by activating persulfate or peroxymonosulfate (PMS) using UV, heat, transition metals, and an alkaline medium (Equations (2) and (3)) [15]. The FLP has shown a notable efficacy in applications such as water treatment, where sulfate radicals mainly react via electron transfer with the pollutants [16,17]. Furthermore, hydroxyl radicals can be generated during this treatment (Equation (4)), and no pH adjustment is necessary because the presence of sulfate radicals reduces the pH in the solution (Equation (5)) [18]. The targeted persulfate or PMS concentration usually varies between 0 and 10 mM [19], and the selection of the targeted concentration depends on the composition of wastewater. Thus, although elevated disinfection can be achieved by a low concentration of 0.1 mM using distilled water as the water matrix [20], a concentration of 10 mM is necessary when using complexing matrices, as reported by Rodriguez-Chueca et al. [21] in the disinfection of winery wastewater.

(2) $$S_{2}O_{8}^{-2}+{\mathrm{UV}\;o\;\mathrm{heat}\;o\;\mathrm{Fe}}^{+2}\to 2{\mathrm{SO}}_{4}^{\cdot -}$$

(3) $${\mathrm{HSO}}_{5}^{-}+{\mathrm{UV}\;o\;\mathrm{heat}\;o\;\mathrm{Fe}}^{+2}\to {\mathrm{SO}}_{4}^{\cdot -}+\mathrm{HO}\cdot$$

(4) $${\mathrm{SO}}_{4}^{\cdot -}+{\mathrm{OH}}^{-}\to {\mathrm{SO}}_{4}^{2-}+\mathrm{HO}\cdot$$

(5) $${\mathrm{SO}}_{4}^{\cdot -}+H_{2}O\to {\mathrm{SO}}_{4}^{2-}+H^{+}+\mathrm{HO}\cdot$$

The inactivation processes by sulfate radicals display potential over traditional disinfection methods that form dangerous disinfection by-products [19]. Nevertheless, different knowledge gaps were presently identified in the inactivation of pathogenic microorganisms by sulfate radicals. Thus, further investigation into the influence of operational factors, such as the dosage of disinfectants, catalyst effect, and treatment time for a comprehensive evaluation of the technology, is needed.

Based on the aforementioned approaches to disinfection research, this study focuses on the optimization using simulated wastewater of the different variables involving the processes: precursor concentration (PMS and H_2_O_2_), catalyst concentration (Fe^+2^), and pH in the Fenton process. To achieve this, the central composite design (CCD) is used for designing the steps of the study and response surface methodology (RSM) is used for the modelling and optimization of the disinfection by the FP and FLP of *E. coli* in simulated wastewater. After that, the optimized conditions are applied to real wastewater matrices.

## 2. Materials and Methods

### 2.1. Bacterial Strain Maintenance and Inoculum

The selected bacterium was provided by the Spanish Culture Type Collection (CETC): the strain NCIMB 9483 of *Escherichia coli* CETC 102. The bacteria were stored in 1 mL cryo-vials of Meat Peptone Broth (20% glycerol) in the freezer at −20 °C. In order to activate the colonies, before the experiment, 0.25 mL (0.5% *v/v*) was inoculated in a 250 mL Erlenmeyer with 50 mL sterilized (121 °C 1.5 atm) Meat Peptone Bacteriologic (MPB) medium (10 g/L bacteriological peptone (Panreac AppliChem, Barcelona, Spain), 5 g/L meat extract (Panreac AppliChem), and 5 g/L NaCl (Carlo Erba Reagents, Sabadell, España, 96%)). Then, bacteria were activated and incubated at 37 °C and 80 rpm for 20 h in an orbital shaker (Thermo Electron Corporation, Forma Orbital Shaker, Waltham, MA, USA) until the stationary phase was reached (optical density around 1 at 600 nm). After that, the colonies were harvested in a centrifuge (Sigma Laboratory Centrifuges, 3K18) for 15 min, 8000 rpm, and at 10 °C. This procedure assured a minimum concentration of 10^10^ colony-forming units (CFUs) per mL. Subsequently, the colonies were resuspended in 5 mL of sterile saline solution 0.9% *w/w*. All materials and pre-prepared solutions for the *E. coli* experiments were sterilized in an autoclave Presoclave II (J.P. Selecta^®^, Barcelona, Spain). The working conditions were 121 °C temperature, 1 bar pressure, and a cycle duration of 20 min.

### 2.2. Disinfection Assays

In all the tests, the disinfection was evaluated at two different treatment times (5 and 15 min), using an effective volume of reaction of 100 mL. Assays were conducted in duplicate and control assays were needed during each run. Synthetic wastewater, where the optimization of both treatments was done, was prepared according to Aldrovandi et al. [22]. Real WWTP samples, where the optimized treatments were studied, were taken from the effluents from secondary and primary treatment at local WWTPs located in Guillarei (Tui, Galicia, Spain). The samples were kept in the fridge at 4 °C and they were used between 1 and 2 weeks later. In the Supplementary Materials, the characterization of these real samples is summarized in Table S1.

#### 2.2.1. FP Disinfection Assays

During the FP, three parameters were evaluated: concentration of H_2_O_2_, concentration of Fe^+2^, and pH. To set up the experiment, the pH was initially adjusted before the wastewater sterilization (121 °C, 20 min). Secondly, 1 mL of the *E. coli* in saline suspension was added to 99 mL of the wastewater in order to inoculate the wastewater (10^10^ CFUs/mL), followed by the catalyst (FeSO_4_ (Panreac AppliChem, 99%)) according to the design of the experiments. Finally, the oxidant hydrogen peroxide (H_2_O_2_ (Sigma-Aldrich, 30%, ≥99.9%)) was added. After that, the flasks were introduced on the orbital shaker (Thermo scientific, MaxQ 8000) at 25 °C, 80 rpm, and under dark conditions. After the disinfection, 1 mL of sample was taken and put into a quench solution of 4% *w/v* Na_2_S_2_O_3_ (Sigma-Aldrich, St. Louis, MO, USA) in 0.25 M KH_2_PO_4_ (Prolabo) for 15 min [23] in order to stop the disinfection. The same procedure was conducted with the control assays to avoid variability due to the effects of the quench solution on the bacteria. The colonies were quantified by the standard plate counting method (CFU method Section 2.4.1).

#### 2.2.2. FLP Disinfection Assays

On the other hand, the FLP had only two variables: PMS and Fe^+2^ concentration, so pH was not adjusted. Similarly, to previous assays, the inoculum, the catalyst (FeSO_4_ (Panreac AppliChem, 99%)), and the oxidant (PMS Sigma-Aldrich) were consecutively introduced in the flask according to the design of the experiments. After that, the flasks were introduced on the orbital shaker (Thermo scientific, MaxQ 8000, Waltham, MA, USA) at 25 °C, 80 rpm, and under dark conditions. After the disinfection, quenching solution was used, and 1 mL of sample was taken and put into a quench solution min (4% *w/v* Na_2_S_2_O_3_ (Sigma-Aldrich) in 0.25 M KH_2_PO_4_ (Prolabo)) for 15 in order to stop the disinfection. Similarly to FP assays, control assays were conducted to avoid variability due to the effects of the quench solution on the bacteria. After that, the colonies were quantified by the standard plate counting method (CFU method Section 2.4.1).

### 2.3. Design of Experiments with Response Surface Methodology

To optimize the disinfection of *E. coli*, a central composite design (CCD) based on a response surface methodology (RSM) was employed in order to estimate the main and interactive effects of parameters on the response [24]. The software Design Expert 7.0.0 (Stat-Ease, Inc., Minneapolis, MN, USA, Windows operating system) was used to establish the experimental matrix and the data analysis. Data were statistically evaluated with multiple R^2^, adjusted R^2^, and noise tests to assess the model adequacy. A *p*-value was used as a tool to check the significance of coefficients. A *p*-value of less than 0.05 indicated that the model or factor was significant.

The ranges of the variables were selected according to the performed pre-tests (screening assays Tables S2 and S3). After that, the different points in the design FP and FLP were selected and are summarized in Table 1 and Table 2, respectively.

The response was evaluated as a dimensionless number in the optimization as follows

(6) $$Y=-\log_{10}\left(\frac{C}{\mathrm{Ci}}\right)$$

where Ci and C are the CFU/mL before and the treatment, respectively

The main reason to use Y was established as a baseline due to magnitude differences that made them incomparable, and this value went between 1–10.

On one hand, in the FP, three factors were evaluated at two levels, so the design was 2^3^. Each experiment was carried out three times and the central point was carried out six times to obtain strong results: the average was made and summarized in Table 3.

On the other hand, in the FLP, two variables at two levels were evaluated, so the design was 2^2^. Same as the FP, in Table 4, all the data are summarized. All the runs were randomly conducted to minimize systematic errors and the systems were fitted using a quadratic equation where β_0_, β_1,_ β_2_ … are constant parameters and X_1,_ X_2_ … represent the system variables (Equation (7))

(7) $$Y\;={\;\beta }_{0}+\overset{k}{\underset{i=1}{\sum }}\beta_{i}X_{i}+\overset{k}{\underset{i=1}{\sum }}\beta_{\mathrm{ii}}X_{i}{}^{2}+\overset{k}{\underset{1\leq i<j}{\sum }}\beta_{\mathrm{ij}}X_{i}X_{j}$$

### 2.4. Analytical Methods

#### 2.4.1. Disinfection Efficiency

To evaluate the efficiency of the disinfection, the samples with the quench solution were diluted, thus creating 10-fold serial dilutions by using buffered peptone water at 15 g/L (Panreac AppliChem). The colonies were quantified by the standard plate counting method (CFU method). In order to measure the concentration of bacterial 100 µL of the dilutions, the colonies were plated in triplicate on MPB-Agar medium (20 g/L agar (Scharlau)) in Petri dishes. Finally, the colonies were counted manually after 24 h and at 37 °C. The detection limit was established between 30 and 300 CFU. Mean count values (of triplicated samples) CFU/mL were obtained and represented, always with a coefficient of variation less than 15%.

#### 2.4.2. Total Organic Carbon and Chemical Oxygen Demand

The characterization of real wastewater was performed initially, and after the treatment for this purpose, total organic carbon (TOC) measurement was conducted, employing an Analytik Jena multi N/C 3100^®^ coupled to a non-dispersive infrared (NDIR) detector (CACTI, Vigo) (Jena, Germany) and chemical oxygen demand (COD) was measured on a UV-spectrophotometer (DR2800, Hach Lange; Düsseldorf, Germany) with LCK 514 cuvette tests, according to official protocol standards DIN 38409-H41-H44 and ISO 6060-1989.

From these results, the TOC and COD removal percentages were determined according to Equations (8) and (9).

TOC removal (%) = (TOC_0_ – TOC_t_)/TOC_0_ × 100 (8)

where TOC_0_ is the initial value before treatment (mg/L) and TOC_t_ is the TOC at the final treatment time (mg/L).

COD removal (%) = (COD_0_ – COD_t_)/COD_0_ × 100 (9)

where COD is the initial value before treatment (mg/L) and COD_t_ is the COD at the final treatment time (mg/L).

## 3. Results and Discussion

### 3.1. Preliminary Assays

Firstly, several experiments were carried out to know the working range of the different parameters involving both disinfection processes (Tables S2 and S3). These experiments were crucial in order to estimate the limits to the studied variables in the CCD. The assays were made without the catalyst, Fe^+2^, to evaluate the capacity of the oxidant and establish the efficiency of treatment time. The concentration of H_2_O_2_ at 294.11 mM was enough for the total disinfection at 5 min. However, the disinfection at low concentrations, 132.36 or 44.12 mM, demonstrated that the presence of the catalyst was required because after 15 min, the disinfection was low. Evaluating the results in Table S2, 44.12 mM H_2_O_2_ was introduced as the bottom limit in the CCD for FP. Basically, analyzing those results, time reaction was established in 15 and 5 min. Thus, the working range was established between 44.12 mM and 132.36 mM to obtain a symmetric matrix.

The same procedure was carried out in the selection of oxidant concentration for the FLP (Table S3): a concentration between 1 mM and 30 mM PMS was tested for 5 and 15 min without the catalyst. Based on the obtained results, the concentration of 1 mM was selected as the lower value and the highest at 5 mM.

### 3.2. CCD Experiments

In this study, the effects of the main factors for the FP and FLP were investigated in *E. coli* inactivation. Based on the preliminary assays, the selection of the levels of the independent variables for both processes was accomplished (Table 1 and Table 2).

For both processes, a similar concentration of catalyst was selected (0~0.58 mM). In the case of FP, the influence of the solution pH in the disinfection process was also evaluated (3–7). According to CCD design, 16 runs for the FP and 10 runs for the FLP were required in order to complete the experiment design. The batch runs were executed and the values of response at 5 and 15 min were presented in Table 3 and Table 4 for the FP and the FLP, respectively.

The same pattern was observed for both processes: at 15 min, the disinfection had higher efficacy than at 5 min; therefore, high exposure time lead to a higher number of radicals being produced. The experimental values of experiments (Table 3 and Table 4) were inputted to Design Expert 7.0.0. software.

The ANOVA analysis was carried out because it is considered a reliable method to conduct the analysis of diagnostic plots, such as the normal probability plot of residuals, and to predict versus actual values and to validate the adequacy of the model [24]. Statistical analyses of the models were performed to determine the variance for the quadratic polynomial model and linear regression coefficients, which indicated that they were obtained by employing a least square technique to predict the quadratic polynomial model. The results of ANOVA for response at 5 min determined the model of FLP was found not significant (Tables S4 and S5). For this reason, only 15 min responses for the FP and the FLP were evaluated (Table 5 and Table 6).

In both scenarios, a quadratic model was chosen in order to establish the equation with the different variables on both systems: the FP (Equation (10)) and the FLP (Equation (11)).

(10) $$\begin{array}{c} Y\;=-2.463+3.559{\;X}_{1}+10.544{\;X}_{2}+1.396{\;X}_{3}-10.765{\;X}_{1}{\;X}_{2}+0.214{\;X}_{1}{\;X}_{3}-0.451{\;X}_{2}{\;X}_{3}+12.138{\;X}_{1}{}^{2}\\ \;\;\;\;\;\;-2.669{\;X}_{2}{}^{2}-0.172{\;X}_{3}{}^{2}\; \end{array}$$

(11) $$Y\;=-4.202+4.960{\;X}_{4}+22.503{\;X}_{5}-1.547{\;X}_{4}{\;X}_{5}-0.4708{\;X}_{4}{}^{2}-24.367{\;X}_{5}{}^{2}\;$$

R^2^, adjusted R^2^, Fisher’s test (F-value), and *p*-value were used to assess the model adequacy. In the FP, as is shown in Table 5, the model was considered strong enough due to the F-value being 11.814 (higher than 4) and the *p*-value is 0.0035 (<0.05) suggesting that it is statically significant [25]. The R^2^ value (0.947) showed a high correlation between the values calculated by the model and the experimental ones, so the bacteria disinfection could be easily predicted. Between the R^2^_adj_ (0.866) and R^2^_pred_ (0.656), the difference was almost 0.2, so they were still in reasonable agreement. The signal noise was relatively high at 13.4 (>4), which is desirable and can measure the signal to noise ratio [24]. Moreover, all the direct variables of H_2_O_2_, Fe^+2^ concentrations, and pH were reported as significant variables in the system because the *p*-value was <0.05, but not the interactions with others. The quadratic function relationship (Equation (10)) between the disinfection and the three influencing factors for the FP determined that the studied factors were significant, and their influence was followed [H_2_O_2_] > [Fe^+2^] > pH.

On the other hand, for the FLP (Table 6), the model was considered consistent where only the PMS was reported as a significant variable, making a nice prediction on the data obtained in the experiments where the F-value was 10.19 and *p*-value was 0.0215. Considering the R^2^_adj_ (0.836) and R^2^_pred_ (0.494) are slightly different, this showed less correlation between the variables and the disinfection. However, the difference between R^2^ and R^2^_adj_ was less than 0.2 In addition to the FP model, the noise signal was 9.666. In the surface studied for the disinfection (Equation (11)), using the FLP only oxidant concentration was reported as a significant variable, and there were no interactions with other studied variables.

### 3.3. Response Surfaces FP

**Figure 1:**
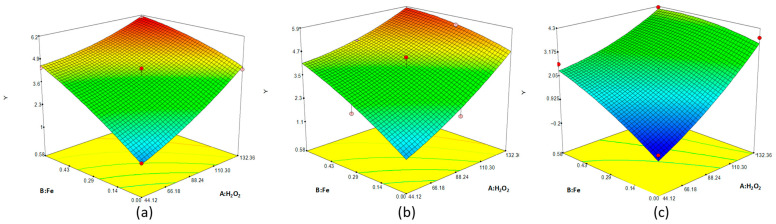
3D response surface of FP showing the reduction colonies (Y) at 15 min in function of the initial concentration of H_2_O_2_ (mM) and Fe^+2^ (mM) at different pHs: (**a**) 3, (**b**) 5 and (**c**) 7.

The 3D plots in Figure 1 show the interaction between the oxidant and the catalyst changing the pH from 3 to 7. Apart from the pH, a high concentration of HO^•^ was produced with the increase in the H_2_O_2_ and Fe^+2^ concentration, as is described by the reaction (Equation (1)), achieving longer disinfection ratios as is observed, which is related to longer ratios oxidant/catalyst [26].

According to the three plots, pH greatly influences the other factors. Thus, the iron accessibility becomes a key factor that strongly depends on the pH [27] because at pH 4.5, the Fe^+3^ is precipitated, and the same occurs at pH 7 for Fe^+2^ species [26]. Thus, low pH allows the homogenous FP, while at high pH, iron is precipitated and heterogeneous catalysis takes places, which reduces the efficiency of the inactivation which results in a high generation of HO^•^, allowing bacteria inactivation [11]. In addition, high pH has a negative influence on H_2_O_2_ producing its decomposition [27], which reduces the process efficiency. Based on the highest ratio, pH 7 shows the worst results, whereas pH 5 and 3 show little difference, which was expected since pH 3 has been cited as the optimum [11,26,27].

### 3.4. Response Surfaces FLP

**Figure 2:**
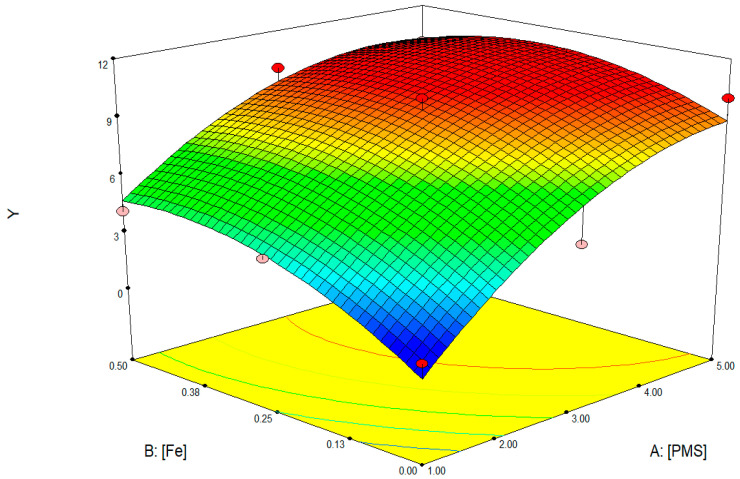
3D response surface of FLP showing the reduction colonies (Y) at 15 min in function of the initial concentration of PMS (mM) and Fe^+2^ (mM).

Comparing the FLP with the FP, the results are really promising because the same reduction levels were reached with low oxidant concentration. According to Xiao et al. [20], SO_4_**^.^**^−^ and HO^•^ can be generated following (Equations (3) and (4)) in the FLP: both radicals are able to oxidize biomolecules such as lipids, carbohydrates, or proteins, achieving bacteria inactivation in a shorter treatment time in comparison to the FP. The response surface depicted in Figure 2 demonstrates the catalyst, in the studied concentrations, has a low impact on the disinfection. The high disinfection was driven by high PMS concentrations because more radicals were generated. It is highlighted that the PMS alone is enough to remove the colonies, so it might also be achieved by the activation of another agent, such as the temperature, as reported in previous studies [21]. PMS can also oxidize some organics directly, without involving radical species [28].

### 3.5. Optimization and Validation in Real Wastewater Plant Matrices

The optimization was conducted by the software Design Expert and the main goal for all the variables was chosen as “in range”, while the target for the Y was selected as “maximize” at 15 min. In the FP, the conditions from the model were 132.36 H_2_O_2_ (mM)/0.56 Fe^+2^ (mM)/3.26 pH, with the expectation to reach 6.052 −Log(C/Ci). In the FLP, the optimized conditions were 3.82 PMS (mM)/0.40 Fe^+2^ (mM), with an expectation to reach 10.641 −Log(C/Ci).

**Figure 3:**
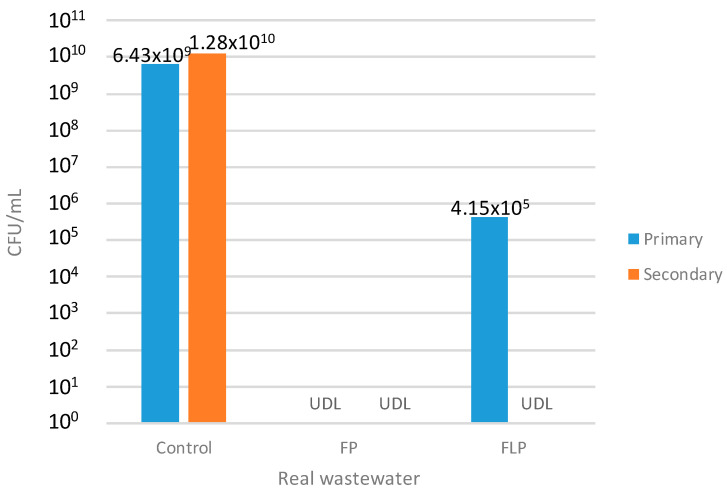
Evaluation of the disinfection in CFU/mL with the optimized conditions on both systems FLP and FP (UDL: under detection limit).

After the optimization, the best conditions for both treatments were tested in real wastewater matrices from the primary and secondary treatment of a WWTP (Table S1). Basically, the FP results are even better than the surface response had predicted because total colonies reduction in the real wastewater was achieved as shown in Figure 3. At the present time, scarce studies are reported in the disinfection, of real effluents and most of the reported process are using combined treatment [21,29]. Therefore, it is interesting to highlight that the obtained results in this study are similar to that obtained by Munoz et al. [30] in the treatment of hospital wastewater by using the Fenton process coupled with thermal treatment (70–90 °C), using a dosage of around 29 mM of H_2_O_2_ after 1 h. Therefore, this fact confirms that the increase of oxidants could be a solution, instead of coupling with costly treatments such as the thermal process, achieving encouraging results in short treatment times.

Meanwhile, in the FLP, the opposite occurred where the reduction was not total in the primary effluent matrix, but complete removal was achieved in the secondary effluent matrix (Figure 3). According to different authors [29], the presence of organic matter and inorganic species in the real matrices can have a negative effect in the AOP. Thus, it is postulated that the presence of nitrate could have a negative effect in the disinfection process by using the FLP by a formation of radicals less active than sulfate radicals [30]. This could explain the reduction in efficiency during the disinfection process. This fact is similar to that previously achieved by Rodriguez–Chueca et al. [21] who reported that, due to the complexity of the water matrix, 10 mM of PMS concentration was required to inactivate microorganisms in real winery wastewaters.

**Figure 4:**
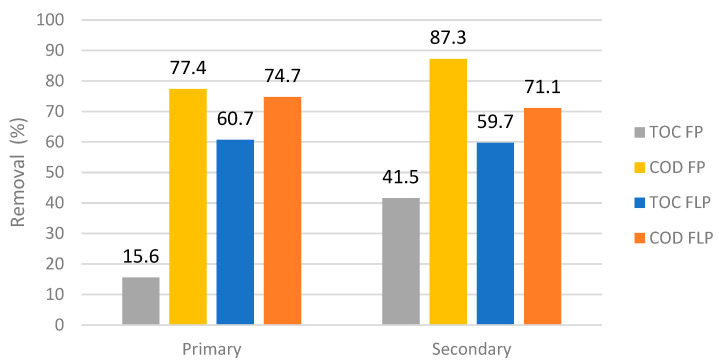
TOC and COD removal (Equations (8) and (9)) after 15 min of treatment in FP and FLP at optimized conditions.

On the other hand, it should be highlighted that both treatments achieved an encouraging result in terms of COD reduction reaching values higher than 70% after 15 min of treatment. In addition, the FLP achieved high TOC removal (around 60%) for both effluents (Figure 4).

## 4. Conclusions

In this study, RSM was utilized to model and optimize the disinfection of *E. coli* process by the FP and the FLP. The obtained models using simulated wastewater were significant and fitted well with the experimental data according to the high values of the coefficient of determination. The quadratic function relationship between the disinfection and the three influencing factors for the FP in *E. coli* disinfection determined that the studied factors were significant, and their influence was followed [H_2_O_2_] > [Fe^+2^] > pH. For the FLP, only [PMS] was reported as significant in the studied space, despite a significant response being obtained by the studied factors, PMS, and Fe dosages. Finally, under optimal conditions, the disinfection efficiency was validated in real wastewater for a WWTP, which demonstrates the viability for the use of these AOP for microbial removal. The present study enhances the knowledge about the use of these technologies, scarcely studied in real matrices, and additionally demonstrates that the optimization of the dosage of oxidants achieved encouraged results similar to the use of combined processes. Therefore, the present study facilitates the practical application of the AOP process in WWTPs.

## Figures and Tables

**Table 1:** Ranges and levels of independent variables for CCD on FP.

Factors	Variables	Levels		
		Low (−1)	Central (0)	High (−1)
[H_2_O_2_] (mM)	X_1_	44.12	88.24	132.36
[Fe^+2^] (mM)	X_2_	0.00	0.29	0.58
pH	X_3_	3	5	7

**Table 2:** Ranges and levels of independent variables for CCD on FLP.

Factors	Symbols	Levels		
		Low (−1)	Central (0)	High (−1)
[PMS] (mM)	X_4_	1.00	3.00	5.00
[Fe^+2^] (mM)	X_5_	0.00	0.29	0.58

**Table 3:** Arrangement of CCD for the three independent variables used in FP.

Run	X_1_	X_2_	X_3_	[H_2_O_2_] (mM)	[Fe^+2^] (mM)	pH	Y_1_ (5 min)	Y_2_ (15 min)
1	−1	−1	−1	44.12	0.00	3	0.246	1.125
2	1	−1	−1	132.36	0.00	3	0.846	4.358
3	0	0	−1	88.24	0.29	3	3.023	4.406
4	−1	1	−1	44.12	0.58	3	3.342	4.437
5	1	1	−1	132.36	0.58	3	4.694	6.016
6	0	−1	0	88.24	0.00	5	1.203	2.295
7	−1	0	0	44.12	0.29	5	1.523	2.422
8	0	0	0	88.24	0.29	5	3.257	4.448
9	0	0	0	88.24	0.29	5	3.257	4.448
10	1	0	0	132.36	0.29	5	4.348	5.470
11	0	1	0	88.24	0.58	5	4.125	4.602
12	−1	−1	1	44.12	0.00	7	0.083	0.152
13	1	−1	1	132.36	0.00	7	0.873	3.861
14	0	0	1	88.24	0.29	7	0.883	1.561
15	−1	1	1	44.12	0.58	7	2.750	2.638
16	1	1	1	132.36	0.58	7	2.809	4.254

**Table 4:** Arrangement of CCD for the three independent variables used in FLP.

Run	X_4_	X_5_	[PMS] (mM)	[Fe^+2^] (mM)	Y_1_ (5 min)	Y_2_ (15 min)
1	0	−1	3	0.00	1.485	4.530
2	−1	1	1	0.50	1.790	4.138
3	0	0	3	0.25	10.000	10.000
4	0	0	3	0.25	10.000	10.000
5	−1	−1	1	0.00	0.000	1.043
6	1	−1	5	0.00	10.000	10.000
7	0	1	3	0.50	10.000	10.000
8	−1	0	1	0.25	0.822	3.806
9	1	0	5	0.25	10.000	10.000
10	1	1	5	0.50	10.000	10.000

**Table 5:** ANOVA results for the response surface quadratic model on the FP process at 15 min.

	Sum of		Mean	F	*p*-Value	
Source	Squares	df	Square	Value	Prob > F	
Model	38.1519	9	4.2391	11.8137	0.0035	significant
X_1_	17.3821	1	17.3821	48.4409	0.0004	significant
X_2_	10.3153	1	10.3153	28.7469	0.0017	significant
X_3_	6.2019	1	6.2019	17.2835	0.006	significant
X_1_X_2_	1.7541	1	1.7541	4.8883	0.0691	
X_1_X_3_	0.0329	1	0.0329	0.0917	0.7722	
X_2_X_3_	0.5471	1	0.5471	1.5248	0.2631	
X_1_^2^	0.1966	1	0.1966	0.5480	0.4871	
X_2_^2^	0.1328	1	0.1328	0.3702	0.5652	
X_3_^2^	1.2527	1	1.2527	3.4911	0.1109	
Residual	2.1530	6	0.3588			
Pure Error	0.0000	1	0			
R^2^	0.947	R^2^_ad_	0.866	R^2^_pred_	0.656	Adeq precision 13.87

**Table 6:** ANOVA results for the response surface quadratic model on FLP process at 15 min.

		Sum of		Mean	F	*p*-Value	
Source	Squares	df	Square	Value	Prob > F		
Model	104.5328	5	20.9066	10.1898	0.0215	significant	
X_4_	73.5910	1	73.5910	35.8681	0.0039	significant	
X_5_	12.2255	1	12.2255	5.9587	0.0711		
X_4_X_5_	2.3945	1	2.3945	1.1671	0.3408		
X_4_^2^	8.2602	1	8.2602	4.0260	0.1153		
X_5_^2^	5.3850	1	5.3850	2.6247	0.1805		
Residual	8.2068	4	2.0517				
Pure Error	0.0000	1	0				
R^2^	0.927	R^2^_ad_	0.836	R^2^_pred_	0.494	Adeq precision	9.666

## Data Availability

Not applicable.

## Supplementary Materials

The following supporting information can be downloaded at: https://www.mdpi.com/article/10.3390/toxics10090512/s1, Table S1: Physical-Chemical characterization on the WWTP (data provided by the WWTP), Table S2: Screening assays for H_2_O_2_ disinfection without catalyst and pH 3, Table S3: Screening assays for PMS without catalyst; Table S4: ANOVA results for the response surface quadratic model on the FP process at 5 min, Table S5: ANOVA results for the response surface quadratic model on the FLP process at 5 min.
