# Supplementary material for: Disinfection through Advance Oxidation Processes: Optimization and Application on Real Wastewater Matrices
Source: Toxics. 2022 Aug 30;10(9):512. doi: 10.3390/toxics10090512 (PMC9501268; doi:10.3390/toxics10090512)
Supplement: Supplementary file 1 [file toxics-10-00512-s001.zip › toxics-1883061-supplementary.pdf]

SUPPLEMENTARY

Table S1 Physical-Chemical characterisation on the WWTP (data provided by the WWTP)

Sample	Cl^{-} (mg/L)	NO_3^{-} (mg/L)	PO_4^{2-} (mg/L)	SO_4^{2-} (mg/L)	TOC (mg/L)	TN (mg/L)	COD (mg/L)	pH	Conductivity (mS/cm)
**Primary effluent**	203.78	67.22	14.1	52.38	51.687	8.063	264	6.06	1.158
**Secondary effluent**	197.738	<0.10	6.332	48.435	18.609	31.999	235	5.61	1.035

Table S2. Screening assays for H_2O_2 disinfection without catalyst and pH 3.

[H_2O_2] (mM)	Time (min)	UFC/mL
0	60	1.76E+10
44.12	15	1.40E+09
132.36	15	8.20 E+05
1	15	0
1	30	0
1	45	0
1	60	0

Table S3 Screening assays for PMS without catalyst

[PMS] (mM)	UFC/mL (5 min)	UFC/mL (15 min)
0.00	1.00E+10	1.00E+10
1.00	2.75E+09	9.05E+07
5.00	0.00	0.00
7.5	0.00	0.00
30.00	0.00	0.00

Table S4 ANOVA results for the response surface quadratic model on the FP process at 5 min ($Y = -5.557 + 4.490\,X_1 + 8.965\,X_2 + 2.359\,X_3 + 0.061\,X_1 X_2 - 0.460\,X_1 X_3 - 0.504\,X_2 X_3 + 2.570\,{X_1}^2 - 2.538\,{X_2}^2 - 0.231\,{X_3}^2$)

Source	Sum of Squares	df	Mean Square	F Value	p-value Prob > F		
Model	31.05	9	3.45	6.48	0.0169	significant	
X_1	3.16	1	3.16	5.94	0.0506		
X_2	20.93	1	20.93	39.31	0.0008		
X_3	2.26	1	2.26	4.24	0.0851		
X_1X_2	5.628E-005	1	5.628E-005	1.057E-004	0.9921		
X_1X_3	0.15	1	0.15	0.29	0.6121		
X_2X_3	0.68	1	0.68	1.29	0.3002		
X_1^2	8.819E-003	1	8.819E-003	0.017	0.9018		
X_2^2	0.12	1	0.12	0.23	0.6517		
X_3^2	2.25	1	2.25	4.23	0.0853		
Residual	3.20	6	0.53				
Pure Error	0.000	1	0.000				
R^2	0.9067	R^2_{ad}	0.7667	R^2_{pred}	0.1215	Adeq precision	8.614

Table S5 ANOVA results for the response surface quadratic model on the FLP process at 5 min ($Y = -7.071 + 5.71\,X_4 + 23.985\,X_5 - 0.895\,X_4\,X_5 - 0.534\,X_4{}^2 - 28.862\,X_5{}^2$)

			**Sum of**		**Mean**	**F**	**p-value**
**Source**	**Squares**	**df**	**Square**	**Value**	**Prob > F**		
Model	165.35	5	33.07	4.42	0.0873	not significant	
X_4	125.02	1	125.02	16.73	0.0150		
X_5	17.70	1	17.70	2.37	0.1987		
$X_4\,X_5$	0.80	1	0.80	0.11	0.7598		
X_4^2	10.64	1	10.64	1.42	0.2988		
X_5^2	7.59	1	7.59	1.02	0.3705		
Residual	29.89	3	9.96				
Pure Error	0.000	1	0.000				
R^2	0.847	R^2_{ad}	0.656	R^2_{pred}	-0.4599	Adeq precision	9.666
